# Predicting First-Semester Performance of First-Year Medical Students in a Changing Landscape

**DOI:** 10.7759/cureus.88463

**Published:** 2025-07-21

**Authors:** Madison Walker, Ashley George, Shane Mohsin, Rhea Nichani, Rahul Garg, Kim Chosie, Christina Kennedy

**Affiliations:** 1 Osteopathic Medicine, Alabama College of Osteopathic Medicine, Dothan, USA; 2 Research, Alabama College of Osteopathic Medicine, Dothan, USA; 3 Medical Education, Alabama College of Osteopathic Medicine, Dothan, USA; 4 Physiology, Alabama College of Osteopathic Medicine, Dothan, USA

**Keywords:** academic performance/grades, assessment in health professions education, grade point average (gpa), mcat, performance indicators

## Abstract

Background

Pre-medical admission factors, such as undergraduate science grade point average (GPA) and the Medical College Admission Test (MCAT), have been extensively studied to predict success in medical school and improve admissions processes. However, the first semester of medical school is critical for student retention and success and is often overlooked by studies that focus on end-of-program success. We analyzed several pre-admission factors to predict student performance on the challenging courses of anatomical sciences and molecular medicine in the first fall semester at an osteopathic medical school.

Materials and methods

This is a retrospective cohort study of 795 doctors of osteopathic medicine (DO) graduates from the 2019-2023 class cohorts. We analyzed the association between pre-medical admission criteria, such as science GPA, MCAT, and undergraduate majors, and performance in anatomy and molecular medicine courses.

Results

Students who met or exceeded the defined scores for competitive admission criteria for undergraduate GPA (p=0.006 and p=0.002), science GPA (both p<0.001), and MCAT total score (p=0.009 and p<0.001) achieved significantly higher grades for both anatomy and molecular medicine courses. Students with biological science majors performed better than those with non-science majors (p=0.002) in the molecular medicine course. Undergraduate majors did not have an association with the performance in the anatomy course.

Conclusions

Our data suggest that GPA, science GPA, MCAT score, and a biology undergraduate degree are significant correlates of improved performance in the challenging first semester of medical school. These findings can help schools identify and support at-risk students during the early, decisive phase of medical education.

## Introduction

Studying in a medical school is a challenging and expensive endeavor. With limited seats per class and rising competition from other schools, medical schools must select students who are likely to succeed on the path to residency. Previous studies have examined several key factors to assess an applicant's academic competitiveness, including undergraduate grade point average (GPA), undergraduate science GPA, Medical College Admission Test (MCAT) scores, and letters of recommendation, to predict the success of a future medical student. For example, a retrospective cohort study found that science test scores were the most reliable predictor of academic performance compared to the other explored admission criteria, accounting for 27.7% of the variation in first-year GPA and 15.0% of the variation in fourth-year GPA. When English test scores and high school GPA were added to the regression analysis, these three factors predicted 40.5% of the first-year GPA and 21.5% of the fourth-year GPA [1]. A study at Boston University's Schools of Medicine and Dental Medicine identified a major in physiology as a positive predictor of success in these graduate programs [2]. Another study demonstrated that students with a pre-admission GPA of less than 3.5 had a significantly higher rate of dropping out of the program (8.3%) compared to their colleagues with a GPA greater than 3.5 (2.9%) from the College of Medicine at King Saud University [3].

Performance during the first semester is crucial in determining a student's retention and success throughout medical school. Students who excel in the first semester and first year tend to continue performing well in the future as well. The first year of the medical school program includes rigorous courses like anatomical sciences and molecular medicine, which challenge the students as the first big "hurdle." A majority of previous studies have examined factors associated with success throughout medical school and on licensure board exams. The MCAT has demonstrated particular predictive value concerning performance on the United States Medical Licensing Examination (USMLE) steps 1 and 2 test scores. Undergraduate science GPA has also been associated with these tests [4-6]. However, the strength of these associations has not been found consistently. For example, one component of the long-term career outcome study found that MCAT performance was only weakly associated with medical school GPA, USMLE step 2 CK, and USMLE step 3 scores. MCAT performance was more strongly associated with USMLE step 1 scores, indicating that it is a more pertinent factor for success on early benchmarks of the medical curriculum. It is essential to determine factors that can predict success during the critical early phase of medical school to identify and tailor the interventions.

We conducted this study to investigate pre-admission factors associated with success in anatomical sciences and molecular medicine courses at an osteopathic medical school. We aim to provide more substantial data on the pre-admission factors that have a strong positive correlation with medical school performance, thereby identifying students who are likely to struggle and may require additional academic support. This data will not only benefit both osteopathic and allopathic medical programs nationwide but also undergraduate programs in guiding their pre-medical students towards academic paths that will increase their chances of getting accepted and succeeding in medical school. It will also help in developing personalized academic assistance to support students who are most at risk of failing their program. Furthermore, we investigated several novel factors with potential predictive power that have not been explored before, particularly among osteopathic medical students. We analyzed the association between undergraduate science GPA, undergraduate GPA, MCAT score, holding a postgraduate degree, the number of MCAT attempts, and undergraduate major and the passing of anatomical sciences and molecular medicine courses.

Disclosure of prior presentation

This article was previously presented as a poster at the 2023 AMA Interim Poster Showcase on November 10, 2023, and at the 2023 ACOM SHM Poster Showcase on December 8, 2023.

## Materials and methods

Design and sample

We conducted a retrospective cohort study following 795 students admitted to the osteopathic medical college. The students' pre-admission records and course performance scores in anatomical sciences and molecular medicine were collected and de-identified before data analysis. Students who were admitted to college between 2015 and 2019 and completed their education by earning a doctorate in osteopathic medicine from the college were included in the study. Students were not required to complete the degree within the standard four-year timeframe to be eligible for the study. Exclusion criteria included failure to graduate or students missing pre-matriculation data points. Additionally, students who withdrew or were dismissed from the program, regardless of the reason or timing of their dismissal, were excluded from the study.

We analyzed the pre-medical factors of undergraduate GPA, undergraduate science GPA, score on first MCAT attempt, total number of MCAT attempts, undergraduate major, and post-baccalaureate education. The pre-admission factors were selected based on existing literature regarding factors that influence academic performance and success in a future medical career. The response variables included performance in anatomical sciences and molecular medicine courses, which are the most challenging and comprise a majority of the academic course load during the first semester at the osteopathic medical school.

The pre-admission factors of undergraduate GPA, undergraduate science GPA, and MCAT score were categorized into "low" and "high" groups, where high was defined as equal to or greater than the competitive admission criteria, as defined by the admissions committee, for the osteopathic medical school: 3.45 for undergraduate GPA, 3.35 for undergraduate science GPA, and 504 for the first MCAT attempt score. The total number of MCAT attempts was analyzed in two categories: one attempt versus multiple attempts. The pre-admission factor of "post-baccalaureate continued education" was categorized based on whether the students enrolled in any form of educational program after completing their undergraduate degree. For the continued education variable, the students were not required to have completed the program in which they enrolled, and the programs included all educational degrees, such as certificates, associate's degrees, master's degrees, and PhDs. These degrees were merged into a single category due to the small sample sizes.

The undergraduate majors were divided into three groups based on the American Medical College Application Service (AMCAS) course classification guide: biological science majors (B), chemistry/physics/math majors (CPM), and all other majors (AO) [7]. Biological science majors included concentrations such as biology, anatomy, ecology, and physiology. The "other majors" included non-science majors such as business, education, psychology, and history. Engineering majors were classified under CPM, despite the AMCAS guide listing engineering under AO, because the engineering curriculum has significant overlap with math and physics and is a core science, technology, engineering, and mathematics (STEM) major.

Statistical methods

We used GraphPad Prism (Dotmatics, Boston, MA, USA), an analytical program, to conduct the statistical analysis. The Shapiro-Wilk test indicated a skewed distribution of the continuous variables for undergraduate GPA, undergraduate science GPA, and scores on anatomical sciences and molecular medicine (p<0.05). Hence, we employed the non-parametric Mann-Whitney U test to determine the association between each pre-admission factor and academic performance in anatomical sciences and molecular medicine. The Kruskal-Wallis test was used to analyze the association between three undergraduate major categories and course performance. We further utilized the Mann-Whitney U test to analyze the bivariate differences in academic performance independently between each undergraduate major group. For all analyses, a p-value of ≤0.05 was considered statistically significant.

## Results

Demographics

Table 1 depicts the demographic composition of each class cohort. Regarding the analyzed study criteria, Table 2 presents the percentage of each cohort that met these criteria. Using the previously reported competitive admissions criteria values for applicants to the studied medical college, students were grouped based on whether they met these criteria and their performance in the fall semester courses of anatomical sciences and molecular medicine in the first year. Additionally, the students were analyzed by their undergraduate major and performance in first-year courses.

**Table 1:** Demographics of the cohorts Demographic data obtained from class profiles maintained by college administration. These demographics represent the total students in each class year, including those who did not meet the inclusion criteria for analysis. For this reason, the total number of students in each class listed here is greater than the one shown in Table 2, which represents the total number of students included in the analysis. GPA: grade point average, MCAT: Medical College Admission Test

Demographics	2019 n=162	2020 n=160	2021 n=161	2022 n=162	2023 n=183
Ethnicity					
White/Caucasian	69.2% (112)	58.1% (93)	49.1% (79)	56.8% (92)	57.4% (105)
Hispanic	7.4% (12)	8.8% (14)	9.9% (16)	9.9% (16)	8.2% (15)
Black/African American	3.7% (6)	5.0% (8)	6.2% (10)	3.7% (6)	4.9% (9)
Asian	14.8% (24)	20.0% (32)	25.5% (41)	24.1% (39)	20.2% (37)
American Indian	0.6% (1)	1.3% (2)	0.0% (0)	0.0% (0)	0.0% (0)
Multiple ethnicities/undefined	4.3% (7)	6.9% (11)	9.30% (15)	5.5% (9)	9.3% (17)
Average GPA	3.4	3.4	3.4	3.38	3.41
Average MCAT	503	503	503	503	504
Female	38% (62)	38% (61)	47% (76)	51% (83)	49% (90)
Male	62% (100)	62% (99)	53% (85)	49% (79)	51% (93)

**Table 2:** Distribution of pre-admission factors Percentage of each cohort that met the criteria to be deemed competitive for the pre-admission factors studied. * "All other" majors include psychology, sociology, economics, political science, public health, and English. Psychology was the most represented major. The datasets for the cohorts were maintained with gaps in student records, resulting in a lower subtotal analysis compared to the total students present in the cohorts. Students who did not have information on one or more of the pre-admission factors were still included in the analysis for any factor for which they had complete data. GPA: grade point average, MCAT: Medical College Admission Test

Pre-admission factors	2019 n=158	2020 n=151	2021 n=159	2022 n=151	2023 n=176
Undergraduate GPA ≥3.45	39% (61/156)	45% (68/151)	42% (66/156)	44% (66/149)	36% (64/176)
Undergraduate science GPA ≥3.35	40% (63/156)	49% (73/151)	39% (61/156)	40% (60/149)	37% (65/176)
First MCAT score ≥504	27% (43/158)	24% (25/106)	30% (47/157)	36% (51/143)	45% (80/176)
Post-baccalaureate continued education	39% (60/155)	38% (54/144)	31% (50/159)	30% (44/149)	39% (69/176)
Undergraduate major					
Biological sciences	57% (83/146)	55% (80/146)	54% (82/153)	64% (95/148)	56% (99/176)
Chemistry, physics, math	8% (12/146)	12% (17/146)	15% (23/153)	11% (16/148)	16% (28/176)
All other*	35% (51/146)	34% (49/146)	31% (48/153)	25% (37/148)	28% (49/176)

Correlation analysis between pre-admission characterization and academic performance

**Figure 1:**
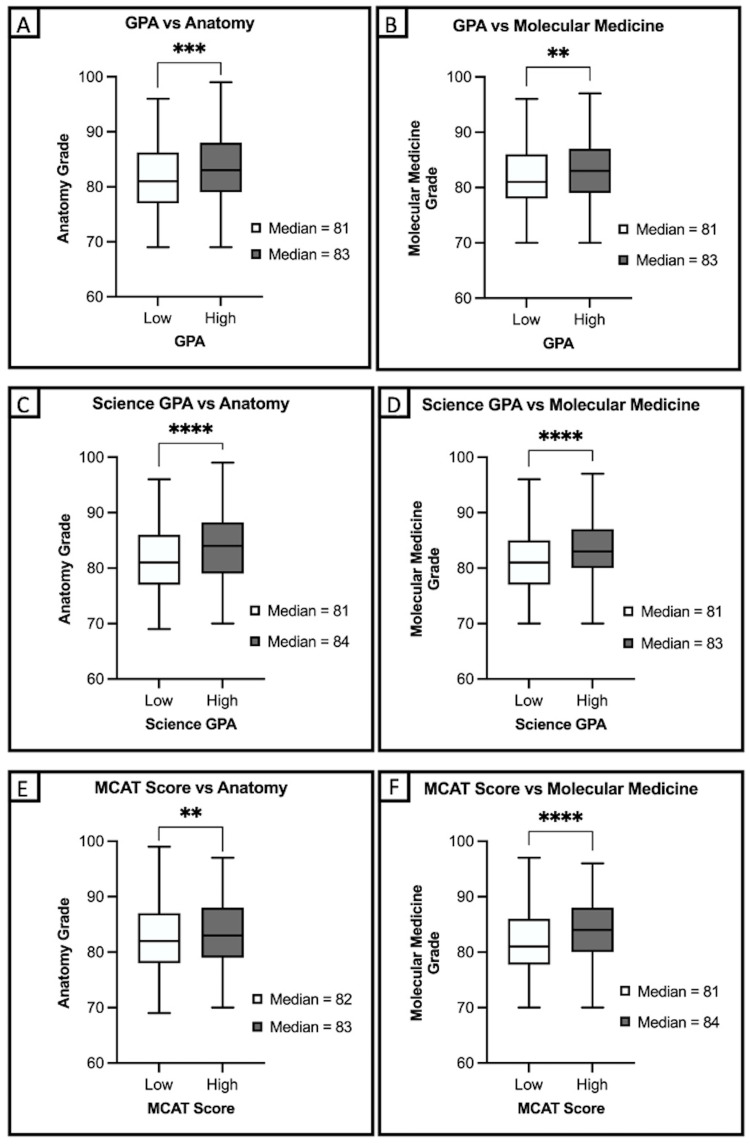
(A-F) Mann-Whitney U analysis of differences in performance on first-year fall semester courses of anatomical sciences and molecular medicine by pre-admission factors, including undergraduate GPA, undergraduate science GPA, and first MCAT score Individuals included in the "high" groups met or exceeded the value of competitive criteria for medical college matriculation for the given factor. *p<0.05*; *p<0.01; ***p<0.001; ****p<0.0001 GPA: grade point average, MCAT: Medical College Admission Test

We found a statistically significant difference in the performance of students in anatomical sciences and molecular medicine courses among those who met or exceeded the competitive criteria for undergraduate GPA, undergraduate science GPA, and first MCAT score, compared to those who did not (Figure 1A-1F). Students with an undergraduate GPA of 3.45 or higher had a median final grade in anatomical sciences of 83. In contrast, those with an undergraduate GPA below 3.45 had a median grade of 81 (p=0.006) (Figure 1A). A similar relationship was observed for the molecular medicine course, with the high undergraduate GPA group scoring a median grade of 83, and the low group achieving a median grade of 81 (p=0.002) (Figure 1B). We observed similar associations between undergraduate science GPA and first MCAT score, with median performance in both courses (Figures 1C-1F). However, we found that the number of MCAT attempts (p=0.89, p=0.15) and post-baccalaureate education (p=0.06, p=0.09) were not significantly associated with median performance in anatomical sciences or molecular medicine.

**Figure 2:**
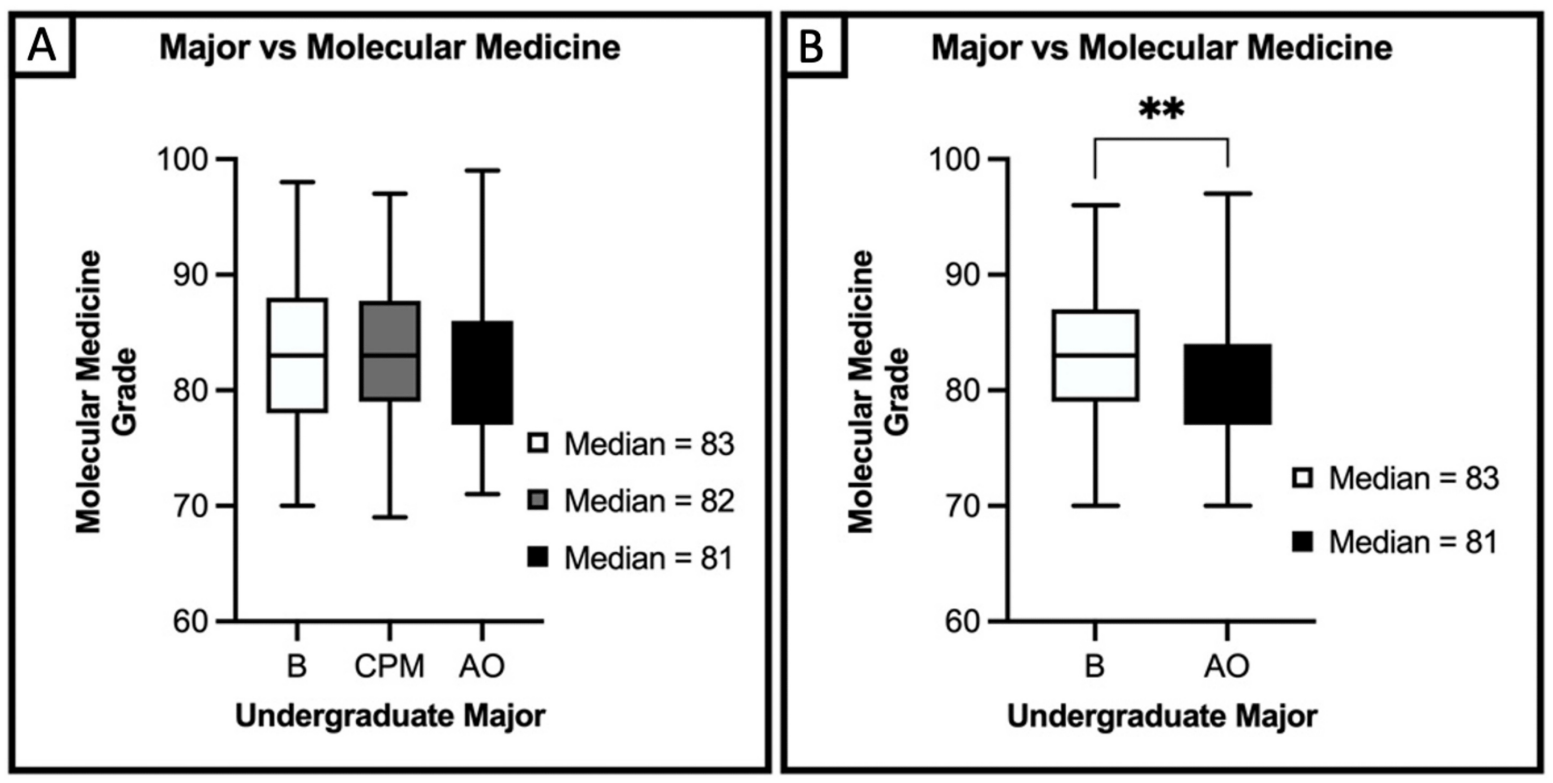
(A) Kruskal-Wallis analysis of performance in molecular medicine by AMCAS undergraduate major classification. (B) Mann-Whitney analysis of groups B and AO demonstrating significant variation No significant differences were noted between groups B and CPM or CPM and AO. AMCAS: American Medical College Application Service, B: biological science majors, CPM: chemistry, physics, and math, AO: all other

Regarding undergraduate majors, we found a statistically significant difference in median scores for molecular medicine between biological science, chemistry/physics/math, and all other majors (p=0.0047) (Figure 2A). Further analysis revealed a significant difference in median performance in molecular medicine between biological sciences and all other undergraduate majors (p=0.002), but no significant difference between chemistry/physics/math and either biology or all other majors (p=0.21, p=0.33) (Figure 2B). We did not find a significant association between undergraduate majors and anatomical sciences (p=0.10).

## Discussion

A possible explanation for why students with biological science majors performed better than their non-STEM peers is the difference in coursework required for degree completion. Biological science degrees often include coursework relevant to molecular medicine, which typically includes only minimal content on anatomy. For example, biochemistry, genetics, cancer cell biology, and neuroscience are all challenging courses that are not required in the pre-medical curriculum but may be required for different biology majors. Although not required for medical school matriculation, these subjects are highly represented in the molecular medicine course. As a result, students with biological science majors likely have some level of pre-exposure to difficult concepts, granting them an advantage over their non-STEM peers. It is reasonable that this advantage does not extend to the medical anatomy course, as that course relies on specific exposure to topics often reserved for the medical school curriculum. At the studied medical college, half of the anatomy course grade is dictated by practical performance in the cadaver dissection lab. Because of this distinction, it is reasonable that we did not find an undergraduate major grouping that conferred an advantage in this course.

The data generated by this study suggest that the number of MCAT testing attempts and whether a student continued their education past their bachelor's degree prior to matriculation had no relationship with the student's performance in the first-year fall semester courses. Due to the limited sample sizes, both factors were divided into two groups. Further analysis with a larger sample size may enable this study to be repeated with a different approach to these two factors, revealing a relationship.

This study contributes to the current understanding of factors influencing medical school success by replicating elements of prior international studies and adjusting components from studies completed on other professional programs. Research on this topic remains scarce, and many studies have been conducted on international medical programs, which have inherent structural and demographic differences from their American counterparts. Additionally, a substantial amount of research on this topic has been conducted in dental schools, which may be more comparable to our research, as it involves American dental students who complete a similar academic pathway to that of medical students. A review of these prior studies finds that results are often inconsistent. In two separate studies, male gender was found to be weakly associated with improved performance, yet also shown to be a risk factor for program failure [8-9]. Depending on the study design, individual courses and time of admission were shown to have varying levels of association with academic performance. However, some factors remained significant across the board and were intentionally included within our study for replication among our student body. Undergraduate GPA and undergraduate science GPA have been consistently shown to correlate positively with various markers of success in higher education. However, the level of association varies from weak to strong [1-3,9-12]. These findings are reflected in our results. In addition to these factors, we also examined the importance of undergraduate major, a concept previously explored in several prior studies [12-13]. Unlike other studies, however, we opted to use the AMCAS major classification guide to group majors for analysis, with the intent of exploring majors using the same lens that medical school admissions committees use. Our findings were inconsistent with both referenced studies because our results demonstrated a significant difference in performance between students with biomedical science majors (B) and non-science majors (AO). This difference may be attributed to our method of grouping majors or the response variables chosen to serve as indicators of medical school success.

Schools can implement this data to provide additional support and resources to newly matriculated students who could potentially be "at risk" based on statistical analysis before or shortly after starting the first year. By proactively addressing any potential weaknesses or concerns, medical schools can continue to holistically evaluate and accept students who may not meet their competitive admissions criteria while ensuring that these students have a high chance of academic success upon matriculation. We hope that this research can inform the development of supportive measures for incoming students who are statistically at risk of underperforming in their first-year fall semester courses. Our stratified analyses can help pinpoint and personalize the support and resources provided to potentially "at-risk" students based on any signs we have identified as "at-risk." For example, as noted, there was a positive correlation between success in the molecular medicine course and matriculation with a major in biological sciences. Therefore, if a student has not completed a degree in biological sciences, there may be gaps in their scientific knowledge base that were not covered in their non-biological sciences major. These gaps can then be addressed by offering summer school courses to bridge the knowledge gaps. In addition to allowing medical schools to address potential weaknesses in "at-risk" students, this data can also be used to educate students about established statistical correlations, making them aware of their risk status regarding future course performance. We believe that if students are aware of the areas where previous classmates with risk factors similar to their own have struggled in the past, they will be more proactive in their academic endeavors and stay especially vigilant in those specific courses or areas of study.

This research can be expanded in future studies by including additional pre-admission characteristics as potential performance indicators, such as age at matriculation, socioeconomic status, hometown population size, parental education level, undergraduate establishment prestige, exposure to relevant coursework in prior education, and learning style. In addition to exploring the relationship between medical course performance and these factors, further research should follow cohorts to investigate whether identified performance indicators remain significant for later milestones, notably second-year courses and USMLE steps 1 and 2. Finally, this research can be further advanced in its application by examining methods that improve performance among students with poor academic outcomes who identify with specific pre-admission factors previously noted within the study. By examining how students who fit specific profiles improve academically, we can begin to design personalized strategies for student assistance that are tailored to each case based on identified risk factors.

Limitations

A survivorship bias is present due to the nature of the data collected for this study. The data were obtained from de-identified records of admitted students enrolled in the medical college between 2014 and 2023; however, only students who completed their studies are represented in this data. Students who withdrew or were dismissed from the program, regardless of the reason or timing of their dismissal, are not present within the dataset. Furthermore, students who did not pass a course and remediated were removed from their original cohort dataset and represented in the next graduating class-year dataset as per administration policy. For these students who repeated a course, the remediated course grades were included in the dataset. As a result, the data used in this study captures only the passing grades of students who have completed the program.

Additional limitations arise from the restriction on the data provided about the students. Information was unidentified, and markers including age, gender, race, and hometown were removed. These factors are present in similar studies on the subject of predictive factors for medical school success. Although the focus of this study was on academic factors, the results could be strengthened by correlating academic outcomes with demographics. In the future, this analysis could be instrumental in supporting at-risk matriculants from underrepresented groups in achieving academic success. Finally, the dataset provided for this research contained missing values, which resulted in the exclusion of data from several individuals from the study.

## Conclusions

Our study revealed a significant correlation between key pre-admission factors and the first-semester academic performance of osteopathic medical students (OMS-I). Analysis revealed that undergraduate GPA, undergraduate science GPA, first MCAT score, and undergraduate major were positive predictors of success in the two challenging courses in the OMS-I fall semester: anatomical sciences and molecular medicine. The increase in course grades for each factor was about 2 points. These results are consistent with existing research in this field on overall performance in the medical schools. Additionally, students with undergraduate biological science majors (including disciplines such as biology, anatomy, ecology, and physiology) performed better than students with non-science majors (business, education, psychology, and history).

## References

[1] Almarabheh A, Shehata MH, Ismaeel A, Atwa H, Jaradat A (2022). Predictive validity of admission criteria in predicting academic performance of medical students: a retrospective cohort study. Front Med (Lausanne).

[2] Davies TA, Miller MB, Moore VA, Kaye EA (2020). Predicting professional school performance with a unique lens: are there other cognitive predictors?. BMC Med Educ.

[3] Al-Mazrou AM (2008). Does academic performance in the premedical year predict the performance of the medical student in subsequent years?. J Family Community Med.

[4] Saguil A, Dong T, Gingerich RJ (2015). Does the MCAT predict medical school and PGY-1 performance?. Mil Med.

[5] Hanson JT, Busche K, Elks ML (2022). The validity of MCAT scores in predicting students’ performance and progress in medical school: results from a multisite study. Acad Med.

[6] Gullo CA, McCarthy MJ, Shapiro JI, Miller BL (2015). Predicting medical student success on licensure exams. BMC Med Sci Educ.

[7] (2023). AMCAS® course classification guide. https://students-residents.aamc.org/applying-medical-school-amcas/amcas-course-classification-guide.

[8] Meyer H, Zimmermann S, Hissbach J, Klusmann D, Hampe W (2019). Selection and academic success of medical students in Hamburg, Germany. BMC Med Educ.

[9] Yates J, James D (2006). Predicting the "strugglers": a case-control study of students at Nottingham University Medical School. BMJ.

[10] Rowland KC, Rieken S (2018). Rethinking dental school admission criteria: correlation between pre-admission variables and first-year performance for six classes at one dental school. J Dent Educ.

[11] Hudson NP, Rhind SM, Mellanby RJ, Giannopoulos GM, Dalziel L, Shaw DJ (2020). Success at veterinary school: evaluating the influence of intake variables on year-1 examination performance. J Vet Med Educ.

[12] Dixon D (2012). Prediction of osteopathic medical school performance on the basis of MCAT score, GPA, sex, undergraduate major, and undergraduate institution. J Am Osteopath Assoc.

[13] Araújo AM, Leite C, Costa P, Costa MJ (2019). Early identification of first-year students at risk of dropping out of high-school entry medical school: the usefulness of teachers' ratings of class participation. Adv Health Sci Educ Theory Pract.

